# Supplementary material for: Toward dimensional psychiatry in youth: A data-driven analysis of transdiagnostic internalizing symptoms in childhood and adolescence
Source: PLOS Ment Health. 2025 Dec 17;2(12):e0000509. doi: 10.1371/journal.pmen.0000509 (PMC12798457; doi:10.1371/journal.pmen.0000509)
Supplement: S1 Text — Table A. Study variables. Overview of variables, scale, and source questionnaires for all variables utilized in FA, MGA, and SEM. Table B. Fit Indices of Three, Four and Eight Factor Solutions. Fit indices of models with an alternative number of factors, i.e., the 3- and 8-factor solutions, are presented here. Table C. Factor Loading Structure of the Four Factor Model. Raw factor loadings of all variables to the factors in the four factor solution of the EFA, in addition to all variable communalities. Table D. Variables with Different Factor Allocation in CFA and Bass-Ackward Analysis. Differences between the CFA and Bass-Ackward method in regards to variable-to-factor allocation. Table E. Additional Fit Indices for MGAs. Reporting of additional fit indices of the MGAs reported in the main manuscript, i.e., strict invariance indices, and fit indices of an additional MGA looking at sex and age combined subgroups (e.g., “male-prepubescent” or “female-postpubescent”). Table F. Regression Effects in SEM. Standardized regression scores between latent factors for all structural models in SEM. Table G. Standardized Regression Coefficients in Piece-Wise SEM of Predictor (Indicator) Analysis. Standardized regression coefficients of the predictor selection process utilizing piece-wise SEM. Table H. Lasso Regression of Predictor (Indicator) Analysis. Standardized regression coefficients of the predictor selection process utilizing lasso regression. Fig A. Imputation of Simulated and Real Missing Values. Density and distribution curves of imputed simulated missing values, imputed real missing values and datasets with missing values and imputed values. Fig B. Path Diagram of the Four Latent Factor Solution. Path diagram with variable-to-factor overview corresponding to the factor loading structure of the four factor solution. (DOCX) [file pmen.0000509.s001.docx]

**Supplementary – S1 Text**

[Section 1. Imputation Strategy. 2](#_Toc214963538)

[Missingness of Data 2](#_Toc214963539)

[Simulation 2](#_Toc214963540)

[Fig A in S1 Text. Imputation on Simulated and Real Missing Values. 2](#_Toc214963541)

[Fig A.1. Density of Original Data and Imputed Values in Simulation. 2](#_Toc214963542)

[Fig A.2. Distribution of simulated imputed data sets and fully observed data set. 5](#_Toc214963543)

[Fig A.3. Distribution of imputed data sets and original data. 6](#_Toc214963544)

[Section 2. Study Variables. 7](#_Toc214963545)

[Table A in S1 Text. Study Variables. 7](#_Toc214963546)

[Section 3. Models with Alternative Number of Factors. 10](#_Toc214963547)

[Table B in S1 Text. Fit Indices of Three, Four and Eight Factor Solutions. 10](#_Toc214963548)

[Section 4. Reasoning for the Four Factor Solution. 11](#_Toc214963549)

[Section 5. Factor Loading Structure of Four Factor Model. 12](#_Toc214963550)

[Table C in S1 Text. Factor Loading Structure of the Four Factor Model. 12](#_Toc214963551)

[Section 6. Path Diagram of Four Factor Model. 14](#_Toc214963552)

[Fig B in S1 Text. Path Diagram of the Four Latent Factor Solution. 14](#_Toc214963553)

[Section 7. Alternative Hierarchical Model (Bass-Ackward Method). 15](#_Toc214963554)

[Table D in S1 Text. Variables with different factor allocation in CFA and Bass-Ackward Analysis. 15](#_Toc214963555)

[Section 8. Fit Indices for Further Model Constraints in MGA and Additional Subsampling MGA. 16](#_Toc214963556)

[Table E in S1 Text. Additional Fit Indices for MGAs. 16](#_Toc214963557)

[Section 9. Regression Effects in SEM. 18](#_Toc214963558)

[Table F in S1 Text. Regression Effects in SEM. 18](#_Toc214963559)

[Section 10. Piece-Wise SEM for Predictor Analysis. 20](#_Toc214963560)

[Table G in S1 Text. Standardized Regression Coefficients in Piece-Wise SEM of Predictor Analysis. 20](#_Toc214963561)

[Section 11. Lasso Regression for Predictor Analysis. 21](#_Toc214963562)

[Table H in S1 Text. Lasso Coefficients of Predictor Analysis. 21](#_Toc214963563)

[References 22](#_Toc214963564)

# Section 1. Imputation Strategy.

## Missingness of Data

In the full HBN data set (N = 4830) with all behavioral and questionnaire variables (N = 6126), the range of missing data per observation is 48% to 91% (mean 60.7%). The range of missing data per variable is 0% to 100%, with some variables having no missing data at all, and others not being used for any observation.

In the subset of selected variables (N = 96), the range of missing data per observations is 0% to 90.6% (mean 11.6%). The range of missing data per variable is 0% to 22%.

In the dataset of observations with less than 30% of missing data (N = 4142, also with 96 variables), the range of missing data per observation is 0% to 29.2% (mean 3%). The range of missing data per variable is 0% to 14.8%. This amount of missing data was deemed as acceptable for imputation.

## Simulation

To check the quality of the imputation methods, a data set of only the fully observed variables was created (N = 2315). From this data set, 11.6% of data were randomly removed. 11.6% corresponds to the mean of missing data per observation of the complete selected variables subset described in the previous section. The selected imputation methods were applied, and the variable distributions of the fully observed data set and the simulated now imputed data set compared. A selection of these comparisons can be found below. In total, the imputation methods recreation of the artificially deleted missing data was very satisfactory and was thus deemed appropriate for the imputation of the missing data for analysis.

## Fig A in S1 Text. Imputation on Simulated and Real Missing Values.

### Fig A.1. Density of Original Data and Imputed Values in Simulation.


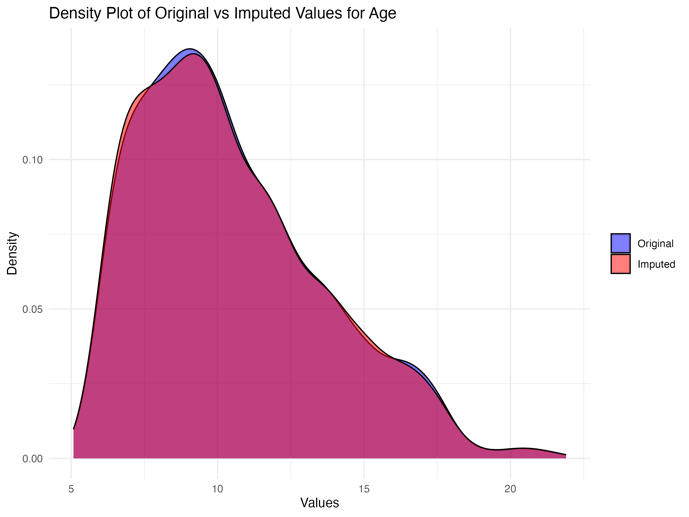

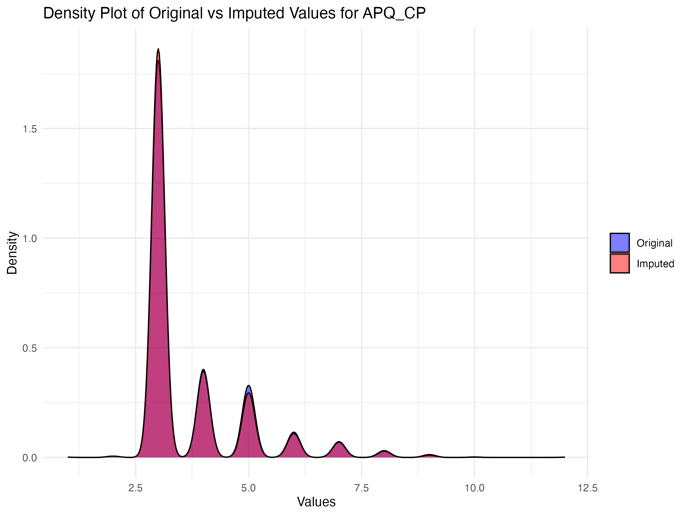


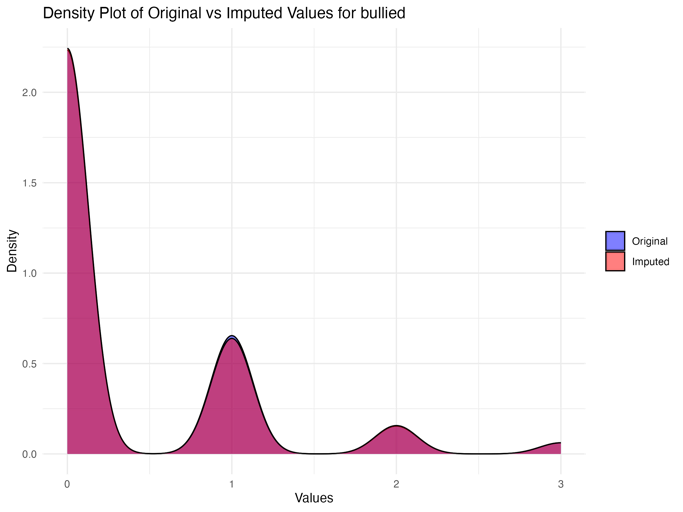

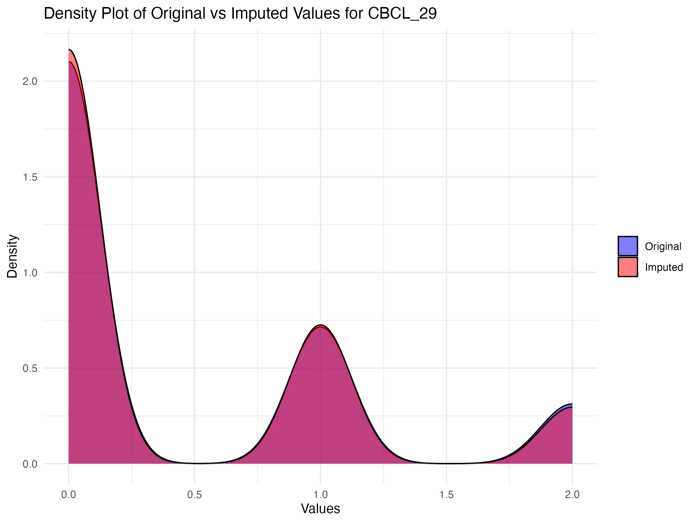

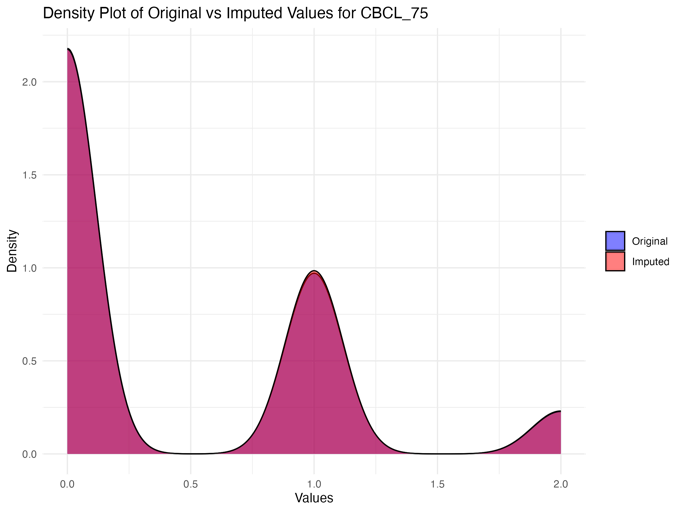

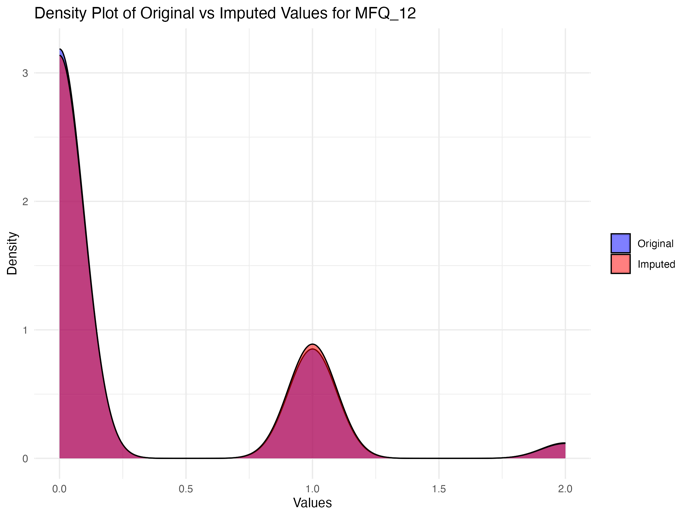

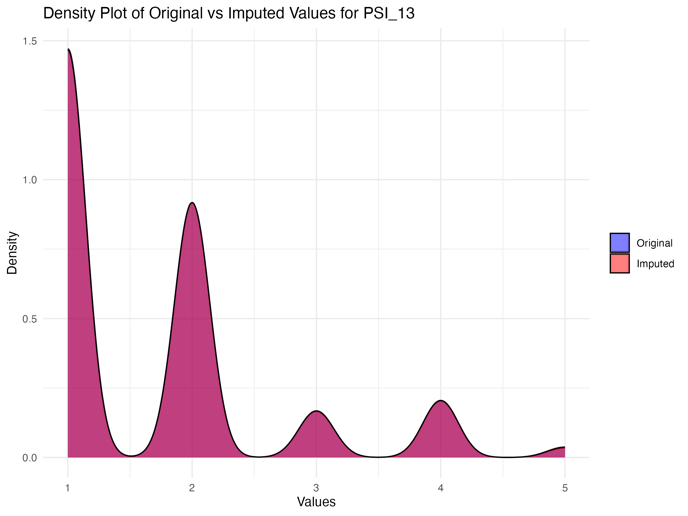

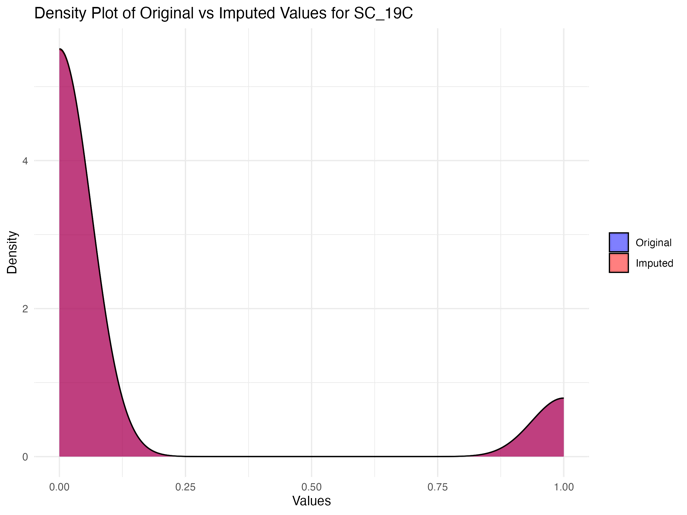

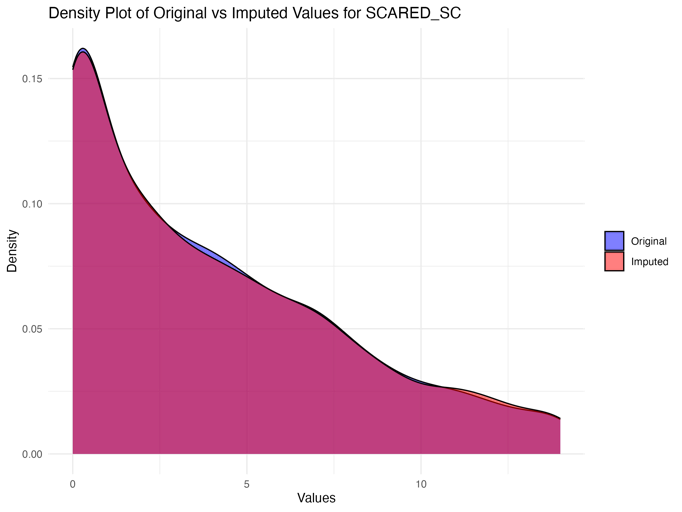

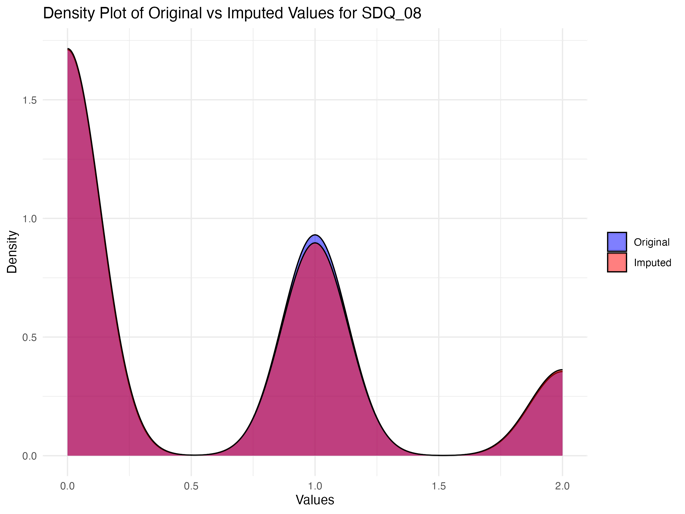

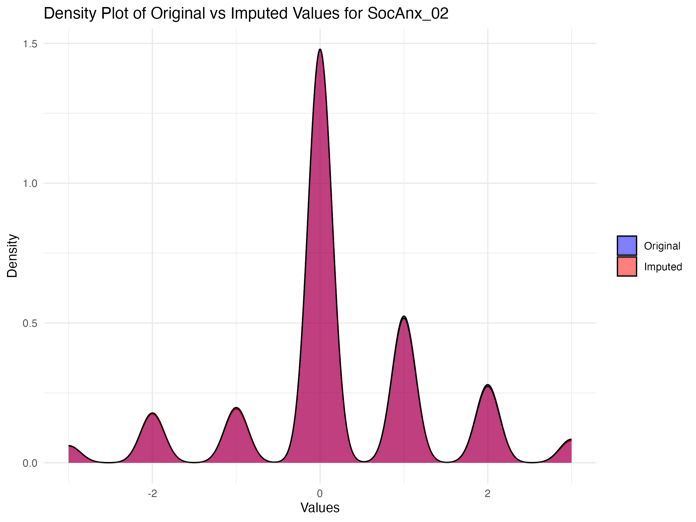

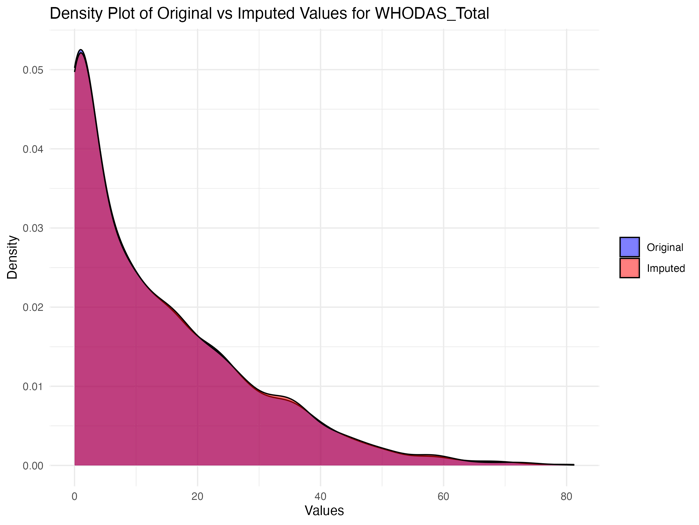


Fig A.2. Distribution of simulated imputed data sets and fully observed data set.


### Fig A.3. Distribution of imputed data sets and original data.

# Section 2. Study Variables.

Variables, Scale, and Source Questionnaires of all variables. The first 78 rows (variables CBCL_05 to SC_33C) are the variables utilized for EFA, CFA and MGA. The next seven rows (variables PSI_07 to APQ_CP) are the predictor variables of the structural equation model, whereas the last row (variable WHODAS_Total) is the outcome variable of the SEM. For copyright reasons, the full variable as presented in the questionnaires to participants cannot be reprinted here. Thus, the second column describes the content of the variables or the construct they represent. These descriptors and labels are sourced mostly from the literature provided in the fourth column. If no such descriptor was available, the variable content was paraphrased. The third column shows the ordinal answer scale of each variable. For example, the scale 0-2 could have the categories 0 = “Not true.”, 1 = “Somewhat or sometimes true”, and 2 = “Very true or often true”.

## Table A in S1 Text. Study Variables.

| **Variable** | **Construct / Content** | **Scale** | **Questionnaire and Literature** |
| --- | --- | --- | --- |
| **CBCL_05** | Enjoys little | 0 - 2 | **Child Behavior Checklist**  Achenbach & Edelbrock, 1983; Dedrick et al., 1997; Gomez & Vance, 2014;  Ivanova et al., 2007; Nakamura et al., 2009  <https://www.apa.org/depression-guideline/child-behavior-checklist.pdf>  <https://aseba.org/aseba-subsequent-developments/> |
| **CBCL_09** | Obsessions | 0 - 2 |  |
| **CBCL_12** | Complains of loneliness | 0 - 2 |  |
| **CBCL_14** | Crying | 0 - 2 |  |
| **CBCL_24** | Not eating well | 0 - 2 |  |
| **CBCL_25** | Animosity | 0 - 2 |  |
| **CBCL_29** | Many fears | 0 - 2 |  |
| **CBCL_33** | Complains about being unloved | 0 - 2 |  |
| **CBCL_35** | Feels worthless | 0 - 2 |  |
| **CBCL_42** | Is rather alone | 0 - 2 |  |
| **CBCL_45** | Nervous | 0 - 2 |  |
| **CBCL_47** | Nightmares | 0 - 2 |  |
| **CBCL_50** | Fearful | 0 - 2 |  |
| **CBCL_53** | Overeating | 0 - 2 |  |
| **CBCL_54** | Overly tired | 0 - 2 |  |
| **CBCL_60** | Genital touching | 0 - 2 |  |
| **CBCL_66** | Repeats actions | 0 - 2 |  |
| **CBCL_68** | Screaming | 0 - 2 |  |
| **CBCL_71** | Self-conscious | 0 - 2 |  |
| **CBCL_73** | Sex problems | 0 - 2 |  |
| **CBCL_75** | Shyness | 0 - 2 |  |
| **CBCL_87** | Mood changes | 0 - 2 |  |
| **CBCL_88** | Sulks | 0 - 2 |  |
| **CBCL_96** | Sex thoughts | 0 - 2 |  |
| **CBCL_100** | Sleep problems | 0 - 2 |  |
| **CBCL_103** | Feels sad | 0 - 2 |  |
| **CBCL_109** | Whining | 0 - 2 |  |
| **CBCL_112** | Worries | 0 - 2 |  |
| **SocAnx_01** | Tolerate feelings of anxiety | -3 - 3 | **Extended Strengths and Weaknesses Assessment of Normal Behaviors**  Alexander et al., 2020  [http://www.eswan.org](http://www.eswan.org/) |
| **SocAnx_02** | Handle fears of seeming anxious | -3 - 3 |  |
| **SocAnx_03** | Stay relaced in social situations | -3 - 3 |  |
| **SocAnx_04A** | Seek out social activities | -3 - 3 |  |
| **SocAnx_04B** | Enjoy social activities | -3 - 3 |  |
| **SocAnx_05** | Control anxiety in social situations | -3 - 3 |  |
| **MFQ_01** | Feels miserable | 0 - 2 | **Mood and Feelings Questionnaire**  Costello & Angold, 1988;  Burleson-Daviss et al., 2006;  Sund et al., 2001 ;  Thabrew et al., 2018  <https://www.corc.uk.net/outcome-experience-measures/mood-and-feelings-questionnaire-mfq/> |
| **MFQ_02** | Enjoys nothing | 0 - 2 |  |
| **MFQ_03** | Less hungry | 0 - 2 |  |
| **MFQ_04** | Eating more | 0 - 2 |  |
| **MFQ_07** | Restless | 0 - 2 |  |
| **MFQ_10** | Indecisiveness | 0 - 2 |  |
| **MFQ_12** | Talks less than usual | 0 - 2 |  |
| **MFQ_15** | Worries of future | 0 - 2 |  |
| **MFQ_21** | Concentration problems | 0 - 2 |  |
| **MFQ_22** | Worries of bad events | 0 - 2 |  |
| **MFQ_23** | Self-Hatred | 0 - 2 |  |
| **MFQ_27** | Feels lonely | 0 - 2 |  |
| **MFQ_28** | Feels unloved | 0 - 2 |  |
| **MFQ_29** | Has no fun at school | 0 - 2 |  |
| **MFQ_30** | Feels inferior to others | 0 - 2 |  |
| **MFQ_34** | Feels no joy when praised | 0 - 2 |  |
| **SCARED_GD** | Generalized anxiety | 0 - 18 | **Screen for Anxiety Related Disorders**  Behrens et al., 2019;  Birmaher et al., 1997, 1999 |
| **SCARED_SC** | Social anxiety | 0 - 26 |  |
| **SCARED_SH** | School avoidance | 0 - 8 |  |
| **SCARED_SP** | Separation fears | 0 - 16 |  |
| **SDQ_03** | Head & stomach aches | 0 - 2 | **Strengths and Difficulties Questionnaire**  Achenbach et al., 2008;  Goodman, 1997;  Goodman et al., 2010;  Muris et al., 2003; Woerner et al., 2004  [http://sdqinfo.org](http://sdqinfo.org/) |
| **SDQ_06** | Preference for solitude | 0 - 2 |  |
| **SDQ_08** | Many worries | 0 - 2 |  |
| **SDQ_16** | Nervous in new situations | 0 - 2 |  |
| **SDQ_24** | Easily scared | 0 - 2 |  |
| **SC_02C** | Often annoyed | 0 - 1 | **HBN’s Symptom Checker**  <http://childmind.org/symptomchecker/> |
| **SC_03C** | Loss of interests | 0 - 1 |  |
| **SC_08C** | High sex interest | 0 - 1 |  |
| **SC_11C** | Anxiety attacks | 0 - 1 |  |
| **SC_12C** | Agoraphobia | 0 - 1 |  |
| **SC_14C** | Fear of kidnapping | 0 - 1 |  |
| **SC_15C** | Worries about harm to parents | 0 - 1 |  |
| **SC_17C** | Trouble sleeping without parents | 0 - 1 |  |
| **SC_18C** | Afraid to be in room without parents | 0 - 1 |  |
| **SC_19C** | Nervous around people | 0 - 1 |  |
| **SC_20C** | Nervous in front of others | 0 - 1 |  |
| **SC_21C** | Unable to speak in front of others | 0 - 1 |  |
| **SC_22C** | Specific phobias | 0 - 1 |  |
| **SC_23C** | Often worries | 0 - 1 |  |
| **SC_25C** | Unable to relax | 0 - 1 |  |
| **SC_26C** | Intrusive thoughts | 0 - 1 |  |
| **SC_27C** | Compulsions | 0 - 1 |  |
| **SC_30C** | Weight worries | 0 - 1 |  |
| **SC_33C** | Binge-eating | 0 - 1 |  |
| **PSI_07** | Parental life dissatisfaction | 1 - 5 | **Parenting Stress Index**  Abidin, 1990, 2006; Ríos et al., 2022  <https://www.parinc.com/Products/Pkey/333> |
| **PSI_13** | Parents having a negative attitude towards their child | 1 - 5 |  |
| **Bullying** | Peer bullying and victimization | 0 - 3 | **HBN’s Pre-Interview Questionnaire**  <https://fcon_1000.projects.nitrc.org/indi/cmi_healthy_brain_network_old/assessments/pre-int.html> |
| **APQ_S_D** | Involvement of mother in child’s life | 0 - 50 | **Alabama Parenting Questionnaire**  Essau et al., 2006;  Frick, 1991;  Shelton et al., 1996  [https://www.youthcoalition.net/wp-content/uploads/2022/06/ APQ.pdf](https://www.youthcoalition.net/wp-content/uploads/2022/06/%20APQ.pdf) |
| **APQ_ID** | Inconsistent child rearing practices and discipline | 0 - 28 |  |
| **APQ_PM** | Parental poor monitoring of child | 0 - 39 |  |
| **APQ_CP** | Corporal punishment of children by parents | 0 - 12 |  |
| **WHODAS_Total** | Daily functioning | 0 - 100 | **WHO Disability Assessment Schedule**  Üstün et al., 2010  <https://www.who.int/standards/classifications/international-classification-of-functioning-disability-and-health/who-disability-assessment-schedule> |

# Section 3. Models with Alternative Number of Factors.

The method of looking at the “elbow” in a scree plot argued for a three factor solution, whereas Vellicer’s MAP proposed 8 factors. In order to comply with the argument of parsimony, the three factor solution was explored. To exhaust other possible options, the eight factor option was also implemented. In this table, the fit indices of all three implemented solutions – models with three, four or eight latent factors – can be compared. All three factor number options were estimated with ML and WLSMV with the purpose of comparing the aptitude of the estimators for the nature of the data. All indices shown in S2 Table are unscaled, due to ML not delivering scaled results. df = degrees of freedom, p = p-value; CFI = Comparative Fit Index; TLI = Tucker Lewis Index; RMSEA = Root Mean Square Error of Approximation; SRMR = Standardized Root Mean Square Residual.

## Table B in S1 Text. Fit Indices of Three, Four and Eight Factor Solutions.

| **Model** | **Fit Measures** | | | | | | | |
| --- | --- | --- | --- | --- | --- | --- | --- | --- |
|  | **df** | **χ^2^** | ***p*** | **CFI** | **TLI** | **RMSEA** | ***p*** | **SRMR** |
| **3 Factors** |  |  |  |  |  |  |  |  |
| ML | 2772 | 22620.830 | <0.001 | 0.696 | 0.671 | 0.059 | <0.001 | 0.049 |
| WLSMV | 2772 | 16133.857 | <0.001 | 0.975 | 0.973 | 0.049 | 0.999 | 0.072 |
| **4 Factors** |  |  |  |  |  |  |  |  |
| ML | 2697 | 19624.104 | <0.001 | 0.741 | 0.711 | 0.055 | <0.001 | 0.044 |
| WLSMV | 2697 | 12022.282 | <0.001 | 0.983 | 0.981 | 0.041 | 1 | 0.064 |
| **8 Factors** |  |  |  |  |  |  |  |  |
| ML | 2407 | 11407.094 | <0.001 | 0.862 | 0.828 | 0.043 | 1 | 0.030 |
| WLSMV | 2407 | 4886.888 | <0.001 | 0.995 | 0.994 | 0.022 | 1 | 0.044 |

# Section 4. Reasoning for the Four Factor Solution.

In the 3 factor solution, the variable CBCL_60 failed to load on any factor with ML but then loaded on the first factor with WLSMV. This solution presented a very unequal factor loading structure, with more than half of the variables allotted to the first factor in the WLSMV solution (factor 1 had 42, factor 2 had 22, and factor 3 had 14 variables). Additionally, it proved to have a lower cumulative explained variance (44.3%) than the four factor solution.

The four factor solution showed improved model fit for both the ML and WLSMV models, and the allocation of variables to factors was more balanced. This solution explained 47.6% of the data’s variance. Further, the additional factor (in comparison with the three factor solution) differentiated a group of variables from the other factors that was theoretically cohesive, and in turn made the other three factors also more coherent in their content.

The eight factor solution seems to be superior, given the improved fit indices, as well as a cumulative explained variance of 56.6%. Yet, this model did not converge, indicating grave model misspecification. Incidentally, the variable allocation was also somewhat unbalanced, as there were factors with as little as 4 variables, while others included 17 variables. Some of the produced factors did not seem theoretically cohesive, i.e., were not interpretable as constructs. Thus, the eight factor solution was not deemed appropriate for the data at hand and discarded.

# Section 5. Factor Loading Structure of Four Factor Model.

Factor Loading Structure of EFA Solution. Shown here are the loadings of the four factor model that was estimated with WLS (WLSMV is not possible with the EFA function in lavaan). Underlined are the strongest factor loadings per variable, i.e., the variables’ factor allocations. In the right-most column, the communalities are shown, which indicate how much of the variance in an observed variables is explained by the extracted factors. The individual variables’ variance impacts these values, with variables with very little variance showing the tendency of having a small communality.

## Table C in S1 Text. Factor Loading Structure of the Four Factor Model.

| **Variable** | **Distress** | **Nervousness** | **Social Fears** | **OC** | **Communalities** |
| --- | --- | --- | --- | --- | --- |
| **CBCL_05** | 0.518 | -0.132 | 0.223 | 0.107 | 0.374 |
| **CBCL_09** | 0.166 | 0.138 | 0.123 | 0.328 | 0.226 |
| **CBCL_12** | 0.436 |  |  |  | 0.245 |
| **CBCL_14** | 0.220 | 0.111 |  | 0.359 | 0.267 |
| **CBCL_24** | 0.190 |  |  | 0.143 | 0.130 |
| **CBCL_25** | 0.255 | -0.131 | 0.200 | 0.378 | 0.270 |
| **CBCL_29** |  | 0.400 | 0.147 | 0.185 | 0.253 |
| **CBCL_33** | 0.500 |  |  | 0.166 | 0.334 |
| **CBCL_35** | 0.599 | 0.174 |  |  | 0.440 |
| **CBCL_42** | 0.246 | -0.177 | 0.479 |  | 0.290 |
| **CBCL_45** | 0.302 | 0.332 | 0.141 | 0.129 | 0.425 |
| **CBCL_47** |  | 0.433 |  | 0.179 | 0.256 |
| **CBCL_50** | 0.204 | 0.545 | 0.145 |  | 0.531 |
| **CBCL_53** | 0.208 |  |  | 0.241 | 0.104 |
| **CBCL_54** | 0.379 |  | 0.146 |  | 0.201 |
| **CBCL_60** |  |  |  | 0.312 | 0.121 |
| **CBCL_66** |  |  | 0.153 | 0.326 | 0.146 |
| **CBCL_68** | 0.199 |  |  | 0.509 | 0.343 |
| **CBCL_71** | 0.249 | 0.282 | 0.222 |  | 0.326 |
| **CBCL_73** |  |  |  | 0.141 | 0.087 |
| **CBCL_75** |  | 0.192 | 0.415 |  | 0.230 |
| **CBCL_87** | 0.455 |  |  | 0.353 | 0.463 |
| **CBCL_88** | 0.501 |  |  | 0.242 | 0.421 |
| **CBCL_96** | 0.118 |  |  | 0.243 | 0.066 |
| **CBCL_100** | 0.163 | 0.246 |  | 0.122 | 0.177 |
| **CBCL_103** | 0.733 |  |  |  | 0.552 |
| **CBCL_109** |  | 0.169 |  | 0.432 | 0.269 |
| **CBCL_112** | 0.249 | 0.572 |  |  | 0.556 |
| **SocAnx_01** |  | 0.152 | 0.711 |  | 0.783 |
| **SocAnx_02** |  | 0.146 | 0.739 |  | 0.761 |
| **SocAnx_03** |  |  | 0.751 |  | 0.630 |
| **SocAnx_04A** |  | -0.133 | 0.824 |  | 0.728 |
| **SocAnx_04B** |  | -0.171 | 0.802 |  | 0.690 |
| **SocAnx_05** |  |  | 0.744 | 0.116 | 0.684 |
| **MFQ_01** | 0.587 |  |  | 0.130 | 0.411 |
| **MFQ_02** | 0.604 |  |  |  | 0.379 |
| **MFQ_03** | 0.266 |  |  |  | 0.130 |
| **MFQ_04** | 0.168 |  |  | 0.241 | 0.131 |
| **MFQ_07** | 0.238 |  |  | 0.370 | 0.238 |
| **MFQ_10** | 0.294 |  |  | 0.166 | 0.201 |
| **MFQ_12** | 0.503 |  |  |  | 0.235 |
| **MFQ_15** | 0.669 |  |  |  | 0.385 |
| **MFQ_21** | 0.353 |  |  | 0.204 | 0.212 |
| **MFQ_22** | 0.371 | 0.333 |  |  | 0.290 |
| **MFQ_23** | 0.630 | 0.121 |  |  | 0.378 |
| **MFQ_27** | 0.562 |  |  |  | 0.329 |
| **MFQ_28** | 0.553 |  |  | 0.162 | 0.368 |
| **MFQ_29** | 0.511 |  |  |  | 0.280 |
| **MFQ_30** | 0.533 | 0.120 |  |  | 0.295 |
| **MFQ_34** | 0.581 |  |  |  | 0.316 |
| **SCARED_GD** | 0.292 | 0.628 | 0.125 | -0.146 | 0.647 |
| **SCARED_SC** |  | 0.322 | 0.542 | -0.144 | 0.430 |
| **SCARED_SH** | 0.317 | 0.338 | 0.103 |  | 0.326 |
| **SCARED_SP** | -0.114 | 0.733 |  | 0.189 | 0.516 |
| **SDQ_03** | 0.232 | 0.222 |  |  | 0.145 |
| **SDQ_06** | 0.163 | -0.187 | 0.482 |  | 0.245 |
| **SDQ_08** | 0.245 | 0.596 |  | -0.129 | 0.566 |
| **SDQ_16** |  | 0.378 | 0.372 |  | 0.386 |
| **SDQ_24** |  | 0.640 | 0.147 |  | 0.504 |
| **SC_02C** | 0.389 |  |  | 0.267 | 0.300 |
| **SC_03C** | 0.415 |  |  |  | 0.220 |
| **SC_08C** |  |  |  | 0.166 | 0.060 |
| **SC_11C** |  | 0.476 |  |  | 0.283 |
| **SC_12C** |  | 0.387 | 0.227 |  | 0.214 |
| **SC_14C** | -0.148 | 0.498 |  |  | 0.218 |
| **SC_15C** |  | 0.447 |  |  | 0.201 |
| **SC_17C** | -0.160 | 0.459 |  | 0.224 | 0.237 |
| **SC_18C** | -0.147 | 0.459 | -0.100 | 0.247 | 0.246 |
| **SC_19C** |  | 0.280 | 0.380 |  | 0.294 |
| **SC_20C** |  | 0.281 | 0.346 |  | 0.235 |
| **SC_21C** |  | 0.160 | 0.294 |  | 0.150 |
| **SC_22C** |  | 0.412 |  | 0.116 | 0.190 |
| **SC_23C** | 0.230 | 0.539 |  |  | 0.436 |
| **SC_25C** | 0.278 | 0.320 |  |  | 0.258 |
| **SC_26C** | 0.256 | 0.379 |  |  | 0.282 |
| **SC_27C** |  | 0.128 | 0.185 | 0.195 | 0.135 |
| **SC_30C** | 0.240 | 0.111 |  |  | 0.164 |
| **SC_33C** | 0.175 |  |  | 0.196 | 0.077 |

# Section 6. Path Diagram of Four Factor Model.

Path Diagram of Model *EFA*. Path diagram of the four factor solution including all variable labels. The arrow color as well as thickness indicates the magnitude of the loading. OC = *Obsessions, Compulsions and Sexual Tendencies*; D = *Distress*; SF = *Social Fears*; N = *Nervousness*.

## Fig B in S1 Text. Path Diagram of the Four Latent Factor Solution.


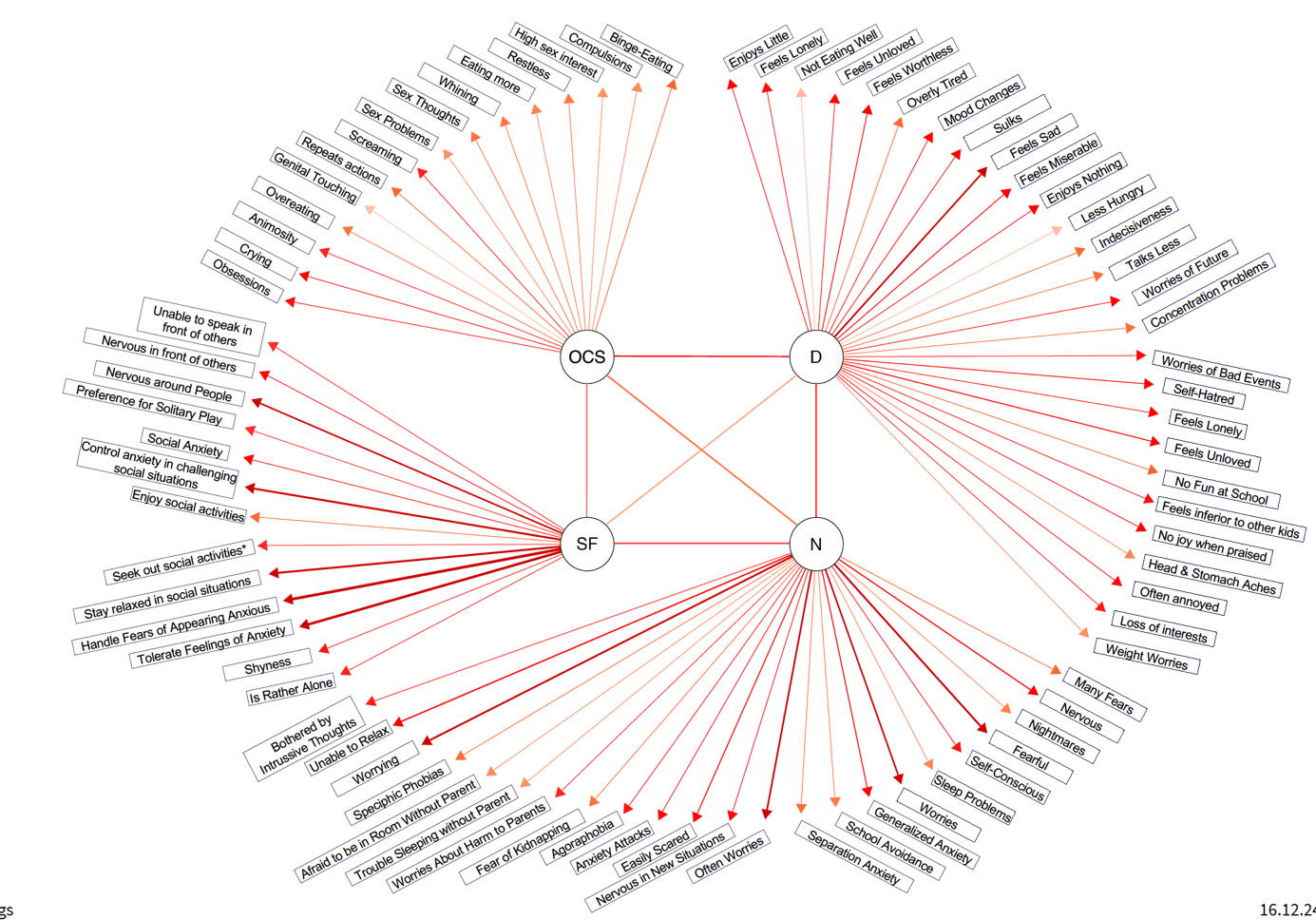


# Section 7. Alternative Hierarchical Model (Bass-Ackward Method).

Differences in Variable-to-Factor allocation in CFA and Bass-Ackward models can be seen in S4 Table. All variable allocation shifts from CFA to the Bass-Ackward approach were to the *Distress* component. The variables still presented high cross-loadings with their original CFA allocation. This may occur due to utilizing different sample sizes (CFA was performed with the second half of the dataset, as the first half was used for EFA, whereas the Bass-Ackward method was implemented using the full dataset). This may also indicate that the structure of internalizing disorders must be further analyzed to confirm and substantiate any findings. Ultimately, this will lead to a robust and valid structure of internalizing disorders for childhood and adolescence. Concludingly, these variable shifts did not fundamentally change the composition and therefore interpretation of the latent factors. The hierarchical component structure extracted by implementing the extended Bass-Ackward method on the complete dataset can be inspected in the main manuscript (Fig 3). The resulting hierarchical structure had an over-arching internalizing component, with second-order factors, i.e., *Distress* and *Fear*, and the factor of *OC* only appearing at the bottom of the hierarchy as a component of *Distress*. *Nervousness* and *Social* *Fears* arose as nodes below *Fear*. Four nodes were recommended for removal due to high redundancy, measured by Pearson correlation and Tucker’s congruence. Specifically on the removal of nodes, b1 and c1 (*Distress* and the node under it) had a correlation of 0.981 and congruence of 0.980, c1 and d1 (the two nodes below *Distress*) had a correlation of 0.960 and congruence of 0.960, c2 and d2 (*Nervousness* and the node below it) had a correlation of 0.982 and congruence of 0.980, and c3 and d3 (*Social Fears* and the node below it) had a correlation of 0.995 and congruence of 0.990. These values approximate 1 and thus indicate the components to (largely) measure the same construct.

## Table D in S1 Text. Variables with different factor allocation in CFA and Bass-Ackward Analysis.

| **Variable** | **Latent Factor** | | | |
| --- | --- | --- | --- | --- |
|  | Distress | Nervousness | Social Fears | OC |
| Nervous | x | x |  |  |
| Eating More | x |  |  | x |
| Restless | x |  |  | x |
| School Avoidance | x | x |  |  |

# Section 8. Fit Indices for Further Model Constraints in MGA and Additional Subsampling MGA.

Fit Indices for Multigroup Analyses. The constraints of equal loadings, intercepts, and residuals (metric, scalar, and error fit respectively) were added. There was little to no difference between the fit of the configural and scalar models for all four MGAs. Interestingly, neither was a difference in fit between the scalar and error models for all MGAs recorded, indicating the model to be quite robust to subgroups. These results also suggest the biggest differences between subgroups being their loadings, as constraining these induced the biggest difference in model fit. All values are scaled.

## Table E in S1 Text. Additional Fit Indices for MGAs.

| **Model** | **Fit Measures** | | | | | | | |
| --- | --- | --- | --- | --- | --- | --- | --- | --- |
|  | **df** | **χ^2^** | ***p*** | **CFI** | **TLI** | **RMSEA [CI]** | ***p*** | **SRMR** |
| **Sex** |  |  |  |  |  |  |  |  |
| Configural | 5836 | 29132.46 | <0.001 | 0.901 | 0.898 | 0.044 [0.043;0.044] | 1 | 0.082 |
| Metric | 5910 | 23959.88 | <0.001 | 0.923 | 0.922 | 0.038 [0.038;0.039] | 1 | 0.083 |
| Scalar | 5989 | 28070.02 | <0.001 | 0.906 | 0.906 | 0.042 [0.042;0.043] | 1 | 0.083 |
| Error | 5993 | 28075.27 | <0.001 | 0.906 | 0.906 | 0.042 [0.042;0.043] | 1 | 0.083 |
| **Age** |  |  |  |  |  |  |  |  |
| Configural | 5836 | 27781.52 | <0.001 | 0.906 | 0.904 | 0.043 [0.042;0.043] | 1 | 0.080 |
| Metric | 5910 | 23267.74 | <0.001 | 0.926 | 0.925 | 0.038 [0.037;0.038] | 1 | 0.082 |
| Scalar | 5989 | 27846.06 | <0.001 | 0.907 | 0.907 | 0.042 [0.041;0.042] | 1 | 0.084 |
| Error | 5993 | 27891.21 | <0.001 | 0.907 | 0.906 | 0.042 [0.042; 0.043] | 1 | 0.084 |
| **Type of Diagnosis** |  |  |  |  |  |  |  |  |
| Configural | 8526 | 26874.09 | <0.001 | 0.907 | 0.907 | 0.039 [0.039;0.040] | 1 | 0.088 |
| Metric | 8672 | 22836.04 | <0.001 | 0.928 | 0.927 | 0.034 [0.034;0.035] | 1 | 0.090 |
| Scalar | 8828 | 25640.07 | <0.001 | 0.915 | 0.915 | 0.037 [0.037;0.038] | 1 | 0.089 |
| Error | 8836 | 25663.05 | <0.001 | 0.915 | 0.915 | 0.037 [0.037;0.038] | 1 | 0.089 |
| **Age-Sex** |  |  |  |  |  |  |  |  |
| Prepub. Male | 2842 | 10007.95 | < 0.001 | 0.922 | 0.919 | 0.039 [0.038;0.040] | 1 | 0.082 |
| Prepub. Female | 2842 | 6708.48 | < 0.001 | 0.914 | 0.912 | 0.039 [0.037;0.040] | 1 | 0.100 |
| Postpub. Male | 2842 | 7548.64 | < 0.001 | 0.905 | 0.903 | 0.041 [0.040;0.042] | 1 | 0.087 |
| Postpub. Female | 2842 | 5257.60 | < 0.001 | 0.932 | 0.930 | 0.038 [0.036;0.039] | 1 | 0.087 |
| Configural | 11368 | 28846.91 | <0.001 | 0.919 | 0.916 | 0.039 [0.038;0.039] | 1 | 0.087 |
| Metric | 11587 | 24968.94 | <0.001 | 0.938 | 0.937 | 0.033 [0.033;0.034] | 1 | 0.090 |
| Scalar | 11821 | 29150.49 | <0.001 | 0.920 | 0.920 | 0.038 [0.037;0.038] | 1 | 0.092 |
| Error | 11833 | 29207.14 | <0.001 | 0.919 | 0.920 | 0.038 [0.037;0.038] | 1 | 0.092 |

# Section 9. Regression Effects in SEM.

Standardized Regression Scores between Latent Factors for Structural Models in SEM. Model A was the full model including all latent factor relationships and did not converge. Hence, no p-values for the regression scores could be computed and fit measures could not be scaled. In models B through G all fit measures are scaled, and p-values could be calculated. Model F was the one selected for further analyses. Model G was a simplified version of model F, but seemed to amalgamate the effect of both *Distress* and *Nervousness* on *OC* within the effect OC ~ D. For purposes of differentiating potentially mediating effects, we decided for the more detailed model F.

## Table F in S1 Text. Regression Effects in SEM.

| **Model** | **Regression Score** | **p-value** | **Fit Measures** |
| --- | --- | --- | --- |
| **A** |  |  |  |
| D ~ N | 0.368 | - | UNSCALED  CFI: 0.962  TLI: 0.960 |
| D ~ SF | -0.135 | - |  |
| D ~ OC | 0.043 | - |  |
| N ~ D | 0.451 | - | RMSEA: 0.058 [0.057;0.058] |
| N ~ SF | 0.070 | - | SRMR: 0.081 |
| N ~ OC | -0.036 | - |  |
| SF ~ D | 0.354 | - |  |
| SF ~ N | 0.431 | - |  |
| SF ~ OC | -0.133 | - |  |
| OC ~ D | 0.464 | - |  |
| OC ~ N | 0.268 | - |  |
| OC ~ SF | 0.077 | - |  |
| **B** |  |  | SCALED  CFI: 0.889; TLI: 0.886  RMSEA: 0.048 [0.047;0.048] |
| D ~ N | 0.341 | < 0.001 |  |
| D ~ SF | 0.084 | < 0.001 |  |
| D ~ OC | 0.463 | < 0.001 | SRMR: 0.079  CFI: 0.889; TLI: 0.886 |
| **C** |  |  |  |
| N ~ D | 0.351 | < 0.001 |  |
| N ~ SF | 0.337 | < 0.001 | RMSEA: 0.048 [0.047;0.048] |
| N ~ OC | 0.236 | < 0.001 | SRMR: 0.079 |
| **D** |  |  |  |
| SF ~ D | 0.132 | < 0.001 | CFI: 0.889; TLI: 0.886 |
| SF ~ N | 0.516 | < 0.001 | RMSEA: 0.048 [0.047;0.048] |
| SF ~ OC | -0.016 | 0.486 | SRMR: 0.079 |
| **E** |  |  |  |
| OC ~ D | 0.533 | < 0.001 | CFI: 0.889; TLI: 0.886 |
| OC ~ N | 0.264 | < 0.001 | RMSEA: 0.048 [0.047;0.048] |
| OC ~ SF | -0.012 | 0.486 | SRMR: 0.079 |
| **F** |  |  |  |
| N ~ D | 0.700 | < 0.001 | CFI: 0.890  TLI: 0.887  RMSEA: 0.048 [0.047;0.048]  SRMR: 0.079 |
| SF ~ N | 0.623 | < 0.001 |  |
| OC ~ D | 0.528 | < 0.001 |  |
| OC ~ N | 0.254 | < 0.001 |  |
| **G** |  |  |  |
| N ~ D | 0.721 | < 0.001 | CFI: 889; TLI: 0.886 |
| SF ~ N | 0.619 | < 0.001 | RMSEA: 0.048 [0.047;0.048] |
| OC ~ D | 0.761 | < 0.001 | SRMR: 0.080 |

# Section 10. Piece-Wise SEM for Predictor Analysis.

Results from piece-wise SEM, testing all predictors on all latent factors simultaneously. Most predictors remained highly significant, albeit some with small effect sizes (e.g. *Distress ~ Age*). The only predictor with a negative effect on all four latent factors seemed to be *Mother Involvement*, potentially indicating a protective effect of higher mother involvement in a child’s life against the development of internalizing psychopathologies. *** <0.001; ** <0.01; * <0.05

The predictors explained some variance of the latent factors. Cumulatively, 21% in the variance of *Distress* was explained by the predictors, 20% of *OC*, 13% of *Nervousness*, and only 7% of *Social Fears*, stipulating that there are other potential risk factors underlying the development of social anxiety and related fears in childhood.

## Table G in S1 Text. Standardized Regression Coefficients in Piece-Wise SEM of Predictor Analysis.

| **Predictor** | **Latent Factor** | | | |
| --- | --- | --- | --- | --- |
|  | **Distress** | **Nervousness** | **Social Fears** | **OC** |
| Age | 0.096 *** | 0.066 *** | 0.110 *** | -0.065 *** |
| Sex | 0.039 ** | 0.072 *** | 0.001 | -0.010 |
| Inconsistent Discipline | 0.185 *** | 0.123 *** | 0.047 ** | 0.208 *** |
| Poor Monitoring | 0.007 | -0.073 *** | -0.054 ** | -0.022 |
| Corporal Punishment | 0.032 * | -0.008 | -0.004 | 0.068 *** |
| Mother Involvement | -0.078 *** | -0.051 *** | -0.056 *** | -0.068 *** |
| Bullying | 0.155 *** | 0.170 *** | 0.118 *** | 0.178 *** |
| Negative Attitude | 0.201 *** | 0.124 *** | 0.141 *** | 0.200 *** |
| Life Dissatisfaction | 0.157 *** | 0.166 *** | 0.089 *** | 0.129 *** |

# Section 11. Lasso Regression for Predictor Analysis.

This method tests all predictors simultaneously, but one latent factor at a time. Similar effects to those with pwSEM were found. Dots (.) signify predictors that were completely regressed to zero. Underlined are predictors kept in the model *SEM ii*. To be kept, an effect size of *β* ≥ 0.1 had to be reached.

## Table H in S1 Text. Lasso Coefficients of Predictor Analysis.

| **Predictor** | **Latent Factor** | | | |
| --- | --- | --- | --- | --- |
|  | **Distress** | **Nervousness** | **Social Fears** | **OC** |
| **Age** | 0.026 | 0.025 | 0.036 | -0.024 |
| **Sex** | 0.071 | 0.187 | . | -0.019 |
| **Inconsistent Discipline** | 0.167 | 0.156 | 0.047 | 0.257 |
| **Poor Monitoring** | 0.006 | -0.089 | -0.046 | -0.024 |
| **Corporal Punishment** | 0.028 | -0.008 | . | 0.083 |
| **Mother Involvement** | -0.069 | -0.064 | -0.060 | -0.081 |
| **Bullying** | 0.139 | 0.217 | 0.135 | 0.219 |
| **Negative Attitude** | 0.182 | 0.158 | 0.164 | 0.247 |
| **Life Dissatisfaction** | 0.142 | 0.212 | 0.101 | 0.159 |

# References

(here in alphabetical order; not numbered in supplementary text)

1. Abidin RR. Parenting Stress Index [Database record]. APA PsycTests; 1990. <https://doi.org/10.1037/t02445-000>
2. Abidin R, Flens JR, Austin WG. The Parenting Stress Index. In: Archer RP, editor. Forensic uses of clinical assessment instruments. Mahwah (NJ): Lawrence Erlbaum Associates Publishers; 2006. p. 297–328.
3. Achenbach TM, Becker A, Döpfner M, Heiervang E, Roessner V, Steinhausen H, et al. Multicultural assessment of child and adolescent psychopathology with ASEBA and SDQ instruments: Research findings, applications, and future directions. J Child Psychol Psychiatry. 2008;49(3):251–75. <https://doi.org/10.1111/j.1469-7610.2007.01867.x>
4. Achenbach TM, Edelbrock C. Manual for the child behavior checklist and revised child behavior profile. Burlington (VT): University of Vermont, Department of Psychiatry; 1983.
5. Alexander LM, Salum GA, Swanson JM, Milham MP. Measuring strengths and weaknesses in dimensional psychiatry. J Child Psychol Psychiatry. 2020;61(1):40–50. <https://doi.org/10.1111/jcpp.13104>
6. Behrens B, Swetlitz C, Pine DS, Pagliaccio D. The Screen for Child Anxiety Related Emotional Disorders (SCARED): Informant discrepancy, measurement invariance, and test–retest reliability. Child Psychiatry Hum Dev. 2019;50(3):473–82. <https://doi.org/10.1007/s10578-018-0854-0>
7. Birmaher B, Brent DA, Chiappetta L, Bridge J, Monga S, Baugher M. Psychometric properties of the Screen for Child Anxiety Related Emotional Disorders (SCARED): A replication study. J Am Acad Child Adolesc Psychiatry. 1999;38(10):1230–6. <https://doi.org/10.1097/00004583-199910000-00011>
8. Birmaher B, Khetarpal S, Brent D, Cully M, Balach L, Kaufman J, et al. The Screen for Child Anxiety Related Emotional Disorders (SCARED): Scale construction and psychometric characteristics. J Am Acad Child Adolesc Psychiatry. 1997;36(4):545–53. <https://doi.org/10.1097/00004583-199704000-00018>
9. Burleson-Daviss W, Birmaher B, Melhem NA, Axelson DA, Michaels SM, Brent DA. Criterion validity of the Mood and Feelings Questionnaire for depressive episodes in clinic and non‐clinic subjects. J Child Psychol Psychiatry. 2006;47(9):927–34. <https://doi.org/10.1111/j.1469-7610.2006.01646.x>
10. Costello EJ, Angold A. Scales to assess child and adolescent depression: Checklists, screens, and nets. J Am Acad Child Adolesc Psychiatry. 1988;27(6):726–37. <https://doi.org/10.1097/00004583-198811000-00011>
11. Dedrick RF, Greenbaum PE, Friedman RM, Wetherington CM. Testing the structure of the Child Behavior Checklist/4-18 using confirmatory factor analysis. Educ Psychol Meas. 1997;57(2):306–13. <https://doi.org/10.1177/0013164497057002009>
12. Essau CA, Sasagawa S, Frick PJ. Psychometric properties of the Alabama Parenting Questionnaire. J Child Fam Stud. 2006;15(5):595–614. <https://doi.org/10.1007/s10826-006-9036-y>
13. Frick PJ. The Alabama Parenting Questionnaire. Unpublished rating scale. Tuscaloosa (AL): University of Alabama; 1991. <https://doi.org/10.1037/t58031-000>
14. Gomez R, Vance A. Confirmatory factor analysis, latent profile analysis, and factor mixture modeling of the syndromes of the Child Behavior Checklist and Teacher Report Form. Psychol Assess. 2014;26(4):1307–16. <https://doi.org/10.1037/a0037431>
15. Goodman R. The Strengths and Difficulties Questionnaire: A research note. J Child Psychol Psychiatry. 1997;38(5):581–6. <https://doi.org/10.1111/j.1469-7610.1997.tb01545.x>
16. Goodman A, Lamping DL, Ploubidis GB. When to use broader internalising and externalising subscales instead of the hypothesised five subscales on the Strengths and Difficulties Questionnaire (SDQ): Data from British parents, teachers and children. J Abnorm Child Psychol. 2010;38(8):1179–91. <https://doi.org/10.1007/s10802-010-9434-x>
17. Ivanova MY, Achenbach TM, Dumenci L, Rescorla LA, Almqvist F, Weintraub S, et al. Testing the 8-syndrome structure of the Child Behavior Checklist in 30 societies. J Clin Child Adolesc Psychol. 2007;36(3):405–17. <https://doi.org/10.1080/15374410701444363>
18. Muris P, Meesters C, Van Den Berg F. The Strengths and Difficulties Questionnaire (SDQ). Eur Child Adolesc Psychiatry. 2003;12(1):1–8. <https://doi.org/10.1007/s00787-003-0298-2>
19. Nakamura BJ, Ebesutani C, Bernstein A, Chorpita BF. A psychometric analysis of the Child Behavior Checklist DSM-Oriented Scales. J Psychopathol Behav Assess. 2009;31(3):178–89. <https://doi.org/10.1007/s10862-008-9119-8>
20. Ríos M, Zekri S, Alonso-Esteban Y, Navarro-Pardo E. Parental stress assessment with the Parenting Stress Index (PSI): A systematic review of its psychometric properties. Children (Basel). 2022;9(11):1649. <https://doi.org/10.3390/children9111649>
21. Shelton KK, Frick PJ, Wootton J. Assessment of parenting practices in families of elementary school-age children. J Clin Child Psychol. 1996;25(3):317–29. <https://doi.org/10.1207/s15374424jccp2503_8>
22. Sund AM, Larsson B, Wichstrøm L. Depressive symptoms among young Norwegian adolescents as measured by the Mood and Feelings Questionnaire (MFQ). Eur Child Adolesc Psychiatry. 2001;10(4):222–9. <https://doi.org/10.1007/s007870170011>
23. Thabrew H, Stasiak K, Bavin L, Frampton C, Merry S. Validation of the Mood and Feelings Questionnaire (MFQ) and Short Mood and Feelings Questionnaire (SMFQ) in New Zealand help‐seeking adolescents. Int J Methods Psychiatr Res. 2018;27(3):e1610. <https://doi.org/10.1002/mpr.1610>
24. Üstün TB, Chatterji S, Kostanjsek N, Rehm J, Kennedy C, Epping-Jordan J, et al. Developing the World Health Organization Disability Assessment Schedule 2.0. Bull World Health Organ. 2010;88(11):815–23. <https://doi.org/10.2471/BLT.09.067231>
25. Woerner W, Fleitlich-Bilyk B, Martinussen R, Fletcher J, Cucchiaro G, Dalgalarrondo P, et al. The Strengths and Difficulties Questionnaire overseas: Evaluations and applications of the SDQ beyond Europe. Eur Child Adolesc Psychiatry. 2004;13(Suppl 2):II47–54. <https://doi.org/10.1007/s00787-004-2008-0>
