# Supplementary material for: Toward dimensional psychiatry in youth: A data-driven analysis of transdiagnostic internalizing symptoms in childhood and adolescence
Source: PLOS Ment Health. 2025 Dec 17;2(12):e0000509. doi: 10.1371/journal.pmen.0000509 (PMC12798457; doi:10.1371/journal.pmen.0000509)
Supplement: S1 Checklist — (DOCX) [file pmen.0000509.s002.docx]

# Questionnaire references (Table 2)

Child Behavior Checklist

Achenbach TM, Edelbrock C. Manual for the child behavior checklist and revised child behavior profile. Burlington (VT): University of Vermont, Department of Psychiatry; 1983.

Dedrick RF, Greenbaum PE, Friedman RM, Wetherington CM. Testing the structure of the Child Behavior Checklist/4-18 using confirmatory factor analysis. Educ Psychol Meas. 1997;57(2):306–13. https://doi.org/10.1177/0013164497057002009

Gomez R, Vance A. Confirmatory factor analysis, latent profile analysis, and factor mixture modeling of the syndromes of the Child Behavior Checklist and Teacher Report Form. Psychol Assess. 2014;26(4):1307–16. https://doi.org/10.1037/a0037431

Ivanova MY, Achenbach TM, Dumenci L, Rescorla LA, Almqvist F, Weintraub S, et al. Testing the 8-syndrome structure of the Child Behavior Checklist in 30 societies. J Clin Child Adolesc Psychol. 2007;36(3):405–17.

https://doi.org/10.1080/15374410701444363

Nakamura BJ, Ebesutani C, Bernstein A, Chorpita BF. A psychometric analysis of the Child Behavior Checklist DSM-Oriented Scales. J Psychopathol Behav Assess. 2009;31(3):178–89. <https://doi.org/10.1007/s10862-008-9119-8>

Extended Strengths and Weaknesses Assessment of Normal Behaviors

Alexander LM, Salum GA, Swanson JM, Milham MP. Measuring strengths and weaknesses in dimensional psychiatry. J Child Psychol Psychiatry. 2020;61(1):40–50. https://doi.org/10.1111/jcpp.13104

Mood and Feelings Questionnaire

Costello EJ, Angold A. Scales to assess child and adolescent depression: Checklists, screens, and nets. J Am Acad Child Adolesc Psychiatry. 1988;27(6):726–37. https://doi.org/10.1097/00004583-198811000-00011

Burleson-Daviss W, Birmaher B, Melhem NA, Axelson DA, Michaels SM, Brent DA. Criterion validity of the Mood and Feelings Questionnaire for depressive episodes in clinic and non-clinic subjects. J Child Psychol Psychiatry. 2006;47(9):927–34. https://doi.org/10.1111/j.1469-7610.2006.01646.x

Sund AM, Larsson B, Wichstrøm L. Depressive symptoms among young Norwegian adolescents as measured by the Mood and Feelings Questionnaire (MFQ). Eur Child Adolesc Psychiatry. 2001;10(4):222–9. https://doi.org/10.1007/s007870170011

Thabrew H, Stasiak K, Bavin L, Frampton C, Merry S. Validation of the Mood and Feelings Questionnaire (MFQ) and Short Mood and Feelings Questionnaire (SMFQ) in New Zealand help-seeking adolescents. Int J Methods Psychiatr Res. 2018;27(3):e1610. <https://doi.org/10.1002/mpr.1610>

Screen for Anxiety Related Disorders

Behrens B, Swetlitz C, Pine DS, Pagliaccio D. The Screen for Child Anxiety Related Emotional Disorders (SCARED): Informant discrepancy, measurement invariance, and test–retest reliability. Child Psychiatry Hum Dev. 2019;50(3):473–82. https://doi.org/10.1007/s10578-018-0854-0

Birmaher B, Brent DA, Chiappetta L, Bridge J, Monga S, Baugher M. Psychometric properties of the Screen for Child Anxiety Related Emotional Disorders (SCARED): A replication study. J Am Acad Child Adolesc Psychiatry.

1999;38(10):1230–6. https://doi.org/10.1097/00004583-199910000-00011

Birmaher B, Khetarpal S, Brent D, Cully M, Balach L, Kaufman J, et al. The Screen for Child Anxiety Related Emotional Disorders (SCARED): Scale construction and psychometric characteristics. J Am Acad Child Adolesc Psychiatry. 1997;36(4):545–53. https://doi.org/10.1097/00004583-199704000-00018

Strengths and Difficulties Questionnaire

Achenbach TM, Becker A, Döpfner M, Heiervang E, Roessner V, Steinhausen H, et al. Multicultural assessment of child and adolescent psychopathology with ASEBA and SDQ instruments: Research findings, applications, and future directions. J Child Psychol Psychiatry. 2008;49(3):251–75. https://doi.org/10.1111/j.1469-7610.2007.01867.x

Goodman R. The Strengths and Digiculties Questionnaire: A research note. J Child Psychol Psychiatry. 1997;38(5):581–6. https://doi.org/10.1111/j.1469-7610.1997.tb01545.x

Goodman A, Lamping DL, Ploubidis GB. When to use broader internalising and externalising subscales instead of the hypothesised five subscales on the Strengths and Difficulties Questionnaire (SDQ): Data from British parents,

teachers and children. J Abnorm Child Psychol. 2010;38(8):1179–91. https://doi.org/10.1007/s10802-010-9434-x

Muris P, Meesters C, Van Den Berg F. The Strengths and Digiculties Questionnaire (SDQ). Eur Child Adolesc Psychiatry. 2003;12(1):1–8. https://doi.org/10.1007/s00787-003-0298-2

Woerner W, Fleitlich-Bilyk B, Martinussen R, Fletcher J, Cucchiaro G, Dalgalarrondo P, et al. The Strengths and Digiculties Questionnaire overseas: Evaluations and applications of the SDQ beyond Europe. Eur Child Adolesc Psychiatry. 2004;13(Suppl 2):II47–54. https://doi.org/10.1007/s00787-004-2008-0
