# Supplementary material for: Toward dimensional psychiatry in youth: A data-driven analysis of transdiagnostic internalizing symptoms in childhood and adolescence
Source: PLOS Ment Health. 2025 Dec 17;2(12):e0000509. doi: 10.1371/journal.pmen.0000509 (PMC12798457; doi:10.1371/journal.pmen.0000509)
Supplement: Attachment — Submitted filename: Engler_ResponseLetter_FA_v4.docx [file pmen.0000509.s004.docx]

Manuscript Number: PMEN-D-25-00214

Title: Toward Dimensional Psychiatry in Youth: A Data-Driven Analysis of Transdiagnostic Internalizing Symptoms in Childhood and Adolescence

Zurich, 23.9.2025

**Re-submission**

Dear Prof. Dr. Cardona,

We would like to thank you and the reviewers for their effort, comments, and suggestions on this manuscript. In order to address all the comments, we have structured the reviewer’s comments and present point-by-point responses below. Based on these excellent comments, we revised the manuscript carefully and included the requested adaptations, which we believe have helped improve the quality of the manuscript.

Following your indications, we have rephrased our “Financial Disclosure Statement” and created a subsection for the “Ethics Statement”, hoping they now adhere to the journal’s submission guidelines. We have also added a legend of the supplementary materials after the bibliography in the main manuscript. We checked the figure formats for compliance with figure requirements and have now also uploaded the manuscript file in .docx format. Regarding the individual comments made by the reviewers, we have quoted and put them in italics, whereas extracts of the main manuscript and adaptations in accordance with the reviewer's remarks are in blue in this rebuttal letter. In accordance with the resubmission guidelines, we have highlighted all changes in yellow in the main manuscript.

We are confident that our adjustments, revisions and comments address all concerns and hope that our manuscript will be suitable for submission in PLOS Mental Health*.*

On behalf of the co-author,

Sincerely,

Ines M. Engler

**Reviewer #1:**

*Your use of a large clinical sample, robust statistical techniques (EFA, CFA, SEM, multi-group analysis), and alignment with HiTOP principles is commendable. However, the manuscript requires major revisions before it can be considered for publication. Below are specific comments to guide your revisions.*

**Comment #1:** *“Strength: You clearly justified the use of factor analysis and model testing across independent datasets. Your statistical approach is rigorous and appropriate, including multi-group invariance testing, the extended Bass-Ackward method, and SEM with regularization. Weakness: Please clarification of variable selection and handling of missing data is needed in the main text. Please briefly explain why 78 variables were retained and summarize the imputation strategy and missingness pattern.”*

**Response #1:**

Thank you for highlighting the need for clarification on variable selection and handling of missing data. We have expanded the Methods section to provide these details. Specifically, we now explain that the 6,126 available behavioral and demographic variables from the HBN were screened for thematic and analytical suitability, as well as for the extent of missing data. The vast majority of variables (~75%) were not related to psychopathological constructs (e.g., physical and bodily measures, study protocol items, age-specific questionnaires, demographic information). Variables were excluded if they (a) did not describe internalizing constructs (i.e., we first identified the relevant questionnaires and then assessed their usability for capturing internalizing constructs), (b) were retrospective, i.e., measure past symptoms, or self-reported, (c) showed insufficient variance (Kaiser-Meyer-Olkin < 0.80), or (d) had more than 30% missing data, among further requisites. This resulted in a final set of 78 parent-reported variables with acceptable distributions and missingness (≤ 22% per variable, mean ≈ 11.6%) and thematic coherence to the HiTOP internalizing subfactors. These variables were drawn from six different questionnaires.

We have prepared a flow chart describing all exclusion steps (Figure 1; see below), and have added a more detailed description of the variable selection process in the revised manuscript:

**Measures** The 6126 available behavioral and demographic variables of the HBN were first screened for thematic and analytical aptitude: variables on physical, bodily and cognitive measures, protocol items, age-specific questionnaires, demographic information (including basic demographics and risk factors), and non-internalizing symptoms and traits, according to their source questionnaires, were first excluded. After, variables with more than 30% of missing data, retrospective and self-report variables were discarded, in addition to variables with too little variance (e.g., high single-category endorsement, i.e., variables that had 85% of answers in the same answer category) and that have similar wording to reduce redundancy (e.g., variables “Cries often” and “Often cries”). Additionally, variables with low sampling adequacy (i.e., their Kaiser-Meyer-Olkin value was < 0.80) [60], and with factor loadings ≤0.20 were removed (see Fig 1). This selection resulted in 78 parent-reported variables with acceptable distributions and missingness (≤22% per variable, mean missingness ≈11.6%) and thematic coherence to the subfactors of the internalizing spectrum as presented in the HiTOP model. The remaining missing data, which was not fully missing at random (i.e., some participants did not receive certain questionnaires if the study clinicians did not deem them necessary) was imputed as described below (see Data Preprocessing). The 78 variables stemmed from six different questionnaires

[…].

**Fig 1. Variable Selection Procedure.** All available HBN variables were screened following data- and content-driven approaches. Variables were sequentially excluded based on different criteria, following broader (e.g., “non-internalizing variables”) to more precise (e.g., “KMO ≤ 0.8) exclusion criteria. The final selection included 78 variables, each one assigned to the four HiTOP subfactors, as well as an additional set of predefined demographic and clinical variables utilized in further analyses, but not in the factor analysis.

We recognize that the screening for thematic and analytical aptitude is somewhat subjective. Accordingly, we have included this point as a limitation in the Discussion:

Our selection of pertinent variables to include in the factor analysis followed a data-driven and a content-driven approach. The former is purely objective, since it removes variables statistically unsuitable for analysis, e.g., the child-reported variables had mostly >80% missing data and were thus deemed non-imputable. The content-driven approach is more subjective, as the exclusion or inclusion of a variable was ultimately (hu)man-made but we tried to make this process as literature driven as possible: We followed the indications of the literature on each variable’s questionnaire concerning the constructs that the variable is intended to measure, and matched these with the constructs included in HiTOP’s internalizing spectrum. For example, if a questionnaire’s documentation explicitly stated the intent of measuring externalizing symptoms, we discarded it.

Regarding the imputation strategy: We used an established multivariate imputation approach, implemented via the mice package in R. The procedure was tailored to the measurement level of the variables (e.g., classification and regression trees for ordinal variables, random forests for variables with many categories, and logistic regression for dichotomous variables). We validated the imputation method through a simulation procedure. For the simulation, we took the subset of the complete dataset that had no missing data whatsoever. From this new subset we randomly removed 11.6% of data, which corresponds to the mean of missing data per observation of the complete dataset. After applying the selected imputation methods (as described above), we compared the distributions of the imputed values with those of the original complete dataset to evaluate how well the methods recovered the true data. The simulation delivered very convincing results, i.e., the distributions remained extremely similar between simulated and original data. Thus, we found the method to be acceptable for the imputation of the “real” missing data. More details on the imputation method can be found in the supplementary, as well as figures showing the overlap of distributions.

We have also clarified this in the Data Preprocessing section of the revised manuscript:

**Data Preprocessing** All analyses were performed in R (version 4.3.1). First, missing data were imputed using a multivariate imputation algorithm (“mice” package in R). For ordinally scaled variables with few categories (i.e., two to six answer categories), classification and regression trees were used as the imputation method, for variables with many categories (i.e., upwards of 8 answer categories) random forests was utilized, and for variables with two categories, logistic regression [62]. We validated the imputation method through a simulation procedure. For the simulation, we randomly removed a percentage of data from the complete dataset, which corresponded to the mean percentage of missing data per observation in the full dataset (~11.6%). These missing values were imputed with the methods described above, and the distributions between imputed and real data compared. The distributional overlap was satisfactory and can be consulted in the “Imputation” section of the supplementary materials. This approach ensured that the retained variables were psychometrically adequate and analytically robust.

**Comment #2:** *“Conceptual clarity: Given the age of the participants, the inclusion of “sexual tendencies” without context may be misinterpreted. We strongly encourage revisiting this label and providing clarification on how these symptoms were defined, measured, and interpreted (OCS-Obsessions, Compulsions, and Sexual Tendencies).”*

**Response #2:** We appreciate this thoughtful comment and understand the concern regarding the potential misinterpretation of the label “sexual tendencies”. The underlying items can be inspected in Figure 2 and in the supplementary materials, and include, for example, “Binge eating” and “Thinks about sex too much,” which we grouped together with obsession and compulsion. Out of the 15 variables subsumed under this factor, four are about sexual symptoms, three on disordered eating, and further three on obsessive compulsive symptoms. This approximately equal distribution was the reason for not reducing the variable name to only one of the domains it represents. Nonetheless, to avoid possible misinterpretation, as well as stigmatizing or pathologizing terminology, we have renamed this factor **“Obsessions and Compulsions”** (OC) in the entirety of the revised manuscript, figures and supplemental materials.

**Comment #3:** *“Practical Implication: How could your model be applied in real-world screening, diagnosis, or intervention planning? This is particularly important for a journal like PLOS Mental Health, which values translational relevance.”*

**Response #3:** Thank you for this valuable insight. We agree that emphasizing translational relevance is important. Our findings highlight several potential pathways for real-world clinical application.

The identified factor structure could inform the design of new age-appropriate screening tools that capture broad internalizing dimensions. Such tools could be implemented efficiently inschools, to identify individuals at elevated risk across domains. In clinical settings, dimensional scores could complement categorical classifications by providing a profile of severity and comorbity, as well as highlighting the symptoms and traits with the biggest burden of disease. For intervention planning, a dimensional model could help tailoring treatments to both shared underlying processes (e.g., emotion regulation strategies across mood disorders) and to the individual patient (e.g., by highlighting the symptoms that need most clinical attention). In this way, our study offers an empirical foundation that can guide the development of age-appropriate screening and diagnostic tools with clear translational value.

We have added a new paragraph to the discussion to address this:

Our findings have practical implications for screening and intervention. The identified dimensional structure can inform the development of new screening tools that capture broad internalizing dimensions. Such tools could serve both clinical and non-clinical settings: For example, in schools they could facilitate early identification of individuals at elevated risk across domains. In clinical settings, dimensional scores could complement categorical classifications by providing a profile of severity and comorbity. Further, in intervention planning, a dimensional model could help tailor treatments to shared underlying processes, as well as to the individual patients. In this way, our study provides an empirical foundation for the development of age-appropriate screening and diagnostic tools with clear translational value.

**Comment #4:** *“Several important details (e.g., variable list, loadings, regression coefficients, fit indices) are only available in the supplementary materials. Please summarize key tables or figures in the main manuscript to improve transparency and reader accessibility.”*

**Response #4:** We appreciate this important suggestion. To improve transparency and accessibility, we have moved summaries of the key information from the supplementary materials into the main manuscript, as well as added a further figure. Specifically, we now provide in the main manuscript (a) a flow chart of the variable selection procedure, indicating how many variables were removed at each step and for which reason(s) (Figure 1), (b) SEM regression coefficients for the final SEM model (Table 5), and (c) the table with all variable IDs, contents, scales and source questionnaires that was originally in the supplementary (before Table S1, now Table 2).

Regarding factor loadings, these are indicated in the heatmap in Figure 2A, as well as variable contents. Fit indices of all models (EFA, CFA, MGAs, SEMs) are listed in Table 3. Full regression coefficients, detailed loadings, and additional robustness checks remain available in the supplementary materials to avoid overburdening the main text. We believe our changes strike a balance between readability and transparency.

**Reviewer #2:**

Very interesting and well-written. Only minor amendments are needed in my view.

**Comment #1:** *“L145 Can you distinguish consent from assent here if applicable”*

**Response #1:** Thank you for pointing this out. We have clarified the consent procedure in the revised manuscript. Specifically, written informed consent was obtained from participants aged 18 years or older. For participants younger than 18, written consent was obtained from their legal guardians, and written assent was obtained from the participants themselves.

Prior to conducting the research, written informed consent was obtained from participants ages 18 or older. For participants younger than 18, written consent was obtained from their legal guardians and written assent obtained from the participant.

**Comment #2:** *“L147 Do you need approval for secondary analysis? If not, make it clearer that the original approval included secondary analyses.”*

**Response #2:** We indicate this under the “Ethics Statement” (new document) and have amended the approval proceedings:

The Healthy Brain Network (HBN) study was approved by the Chesapeake Institutional Review Board (https://www.chesapeakeirb.com/), and the approval explicitly includes permission for secondary data analyses. Data access requires completion of the HBN Data Usage Agreement (DUA), which we obtained prior to conducting the present analyses.

**Comment #3:** *“L150 Classification into internalizing/externalizing as a % rather contradicts the focus on dimensional models. I would expect M (SD) of some z-score or similar here.”*

**Response #3:** Thank you for pointing this out. We agree that reporting percentages may suggest a categorical framing and contradict our dimensional focus. To address this, we now include the mean and standard deviation of the Child Behavior Checklist (CBCL) internalizing and externalizing dimensional scale scores in Table 1 of the revised manuscript (newly added: “Clinical Scores (CBCL)” section). This addition provides a dimensional description of the sample that is consistent with our overall modeling approach. However, we also retained the percentages of participants classified as internalizing/externalizing, as these are based on DSM-oriented diagnostic cutoffs, which are categorical by nature. This way, we provide both a dimensional description of the sample and the conventional categorical information.

Sample characteristics, such as age, sex, race, number of diagnoses and CBCL T-scores, are displayed in Table 1.

| **Measure** | **HBN Sample** | **DS1** | **DS2** |
| --- | --- | --- | --- |
| **N** | 4830 | 2045 | 2097 |
| **Age** |  |  |  |
| Range | 5.004 – 21.899 | 5.055 – 21.899 | 5.080 – 21.510 |
| Mean | 10.31 | 10.38 | 10.35 |
| **Sex** |  |  |  |
| n (%) | ♂3071 (63.58%) | ♂1302 (63.67%) | ♂1333 (63.57%) |
| **Race and Ethnicity** |  |  |  |
| White / Caucasian | 2276 (47.12%) | 1089 (53.25%) | 1060 (50.55%) |
| Black / African American | 587 (12.15%) | 260 (12.71%) | 274 (13.07%) |
| Hispanic | 401 (8.30%) | 185 (9.05%) | 180 (8.58%) |
| Asian | 132 (2.73%) | 58 (2.84%) | 62 (2.96%) |
| Indian | 28 (0.58%) | 8 (0.39%) | 16 (0.76%) |
| Two or more races | 711 (14.72%) | 319 (15.6%) | 350 (16.69%) |
| Unknown or otherwise specified | 695 (14.39%) | 126 (6.16%) | 155 (7.39%) |
| **Clinical Diagnoses (DSM)** |  |  |  |
| n (%) | 3845 (79.61%) | 1772 (86.65%) | 1816 (86.60%) |
| Internalizing (%) | 896 (18.55%) | 407 (19.90%) | 432 (20.60%) |
| Externalizing (%) | 2949 (61.06%) | 1365 (66.75%) | 1384 (66%) |
| No Clinical Diagnosis (%) | 390 (8.07%) | 168 (8.22%) | 168 (8.01%) |
| Incomplete Evaluation (%) | 595 (12.32%) | 105 (5.13%) | 113 (5.39%) |
| **Clinical Scores (CBCL)** |  |  |  |
| Mean Int. T-Score (SD) | 56.60 (11.31) | 56.79 (11.41) | 56.40 (11.24) |
| Mean Ext. T-Score (SD) | 54.97 (11.75) | 55.00 (11.83) | 54.87 (11.63) |
| Mean AP T-Score (SD) | 62.90 (9.77) | 62.94 (9.69) | 62.82 (9.83) |

**Table 1**. Sample Characteristics. Shown are number of observations, age range and mean age, the total and percentual number of male observations, the total and percentual number of different races and ethnicities, and the total and percentual number of diagnoses and diagnostic subgroups. “Clinical Diagnoses” are DSM-based categorical diagnoses, whereas “Clinical Scores” are CBCL-based T-scores. Note that “Externalizing” in the DSM refers to neurocognitive and neurodevelopmental disorders, i.e., ADHD or ASD, in addition to behavioral disorders, whereas the CBCL Externalizing score only includes behavioral, aggressive and disruptive disorders. For comparability, the CBCL’s Attention Problems T-Score is also included, which represents neurodevelopmental disorders. DS1 = Dataset 1; DS2 = Dataset 2; Int = Internalizing; Ext = Externalizing; AP = Attention Problems.

**Comment #4:** *“* *L157 Rewrite "Followingly," - not the right word.”*

**Response #4:** Thank you for the hint:

After, variables describing non-internalizing constructs, retrospective and self-report variables were discarded.

[Note: This sentence was scratched from the manuscript altogether after further changes.]

**Comment #5:** *“L160 The data relies on parental reports, which will reduce reliability vs. teacher/peers and under-estimate some of the behaviours e.g. external, compulsions. This needs greater prominence in the discussion section*.*”*

**Response #5:** We appreciate this important point and agree that relying solely on parental reports introduces bias. In particular, externalizing symptoms may be more salient and thus more readily reported by third parties, whereas internalizing symptoms often require greater sensitivity and careful observation. We had not explicitly addressed the limitation of using parent-reported variables, and we have therefore revised the Discussion to highlight this issue more prominently. In the revised manuscript, we now discuss the potential underestimation of certain behaviors (e.g., compulsions, externalizing symptoms) due to reliance on parental reports and have added references to support this well-documented bias.

As with any factor analysis, the results of this work are influenced by the included variables, i.e., their content and informants. Regarding informants, the present study included variables based on parental reports, which might introduce a systematic bias [108, 109]. Externalizing symptoms are often more salient and therefore more readily reported by parents, whereas internalizing symptoms typically require greater sensitivity and may be underestimated. The under-reporting of internalizing symptoms should be considered when interpreting our findings. Nonetheless, the imbalance between parents-reports in externalizing and internalizing domains is less of a problem here, since we only included internalizing symptoms. Considering variable content, studies examining the internalizing spectrum in adults have identified varying factorial structures depending on the included constructs (for an overview, see [23]). Our selection of pertinent variables to include in the factor analysis followed a data-driven and a content-driven approach. The former is purely objective, since it removes variables statistically unsuitable for analysis, e.g., the child-reported variables had mostly >80% missing data and were thus deemed non-imputable.

**Comment #6:** *“L161 References need for the six questionnaires. Why didn't you use the PID-5 adolescent version?”*

**Response #6:** Thank you for this helpful comment. The PID-5 adolescent version was not used because it is not part of the Healthy Brain Network’s behavioral battery and therefore unavailable for analysis. Instead, we relied on other dimensional symptom measures included in the dataset, such as the CBCL. We have now ensured that all six questionnaires are referenced in the main manuscript, indicating their provenance in the text, and indicating a table with more information on the variables:

The 78 variables stemmed from six different questionnaires (Child Behavior Checklist, Extended Strengths and Weaknesses Assessment of Normal Behaviors, Mood and Feelings Questionnaire, Screen for Anxiety Related Disorders, Strengths and Difficulties Questionnaire, HBN’s Symptom Checker; see Table 2) were selected for factor analysis (FA) […].

**Comment #7:** *“L181 Assuming random stratification? Please state for clarity.”*

**Response #7:** Yes, we confirm that the stratification was random while controlling for age and sex. The revised manuscript now states:

Then, the data were randomly stratified into two halves while controlling for age and sex, resulting in two comparable datasets for EFA and CFA (Dataset 1 (DS1) = 2045 participants; Dataset 2 (DS2) = 2097 participants).

**Comment #8:** *“L230 How was pre/post-pubescent determined?”*

**Response #8:** Thank you for raising this point. We clarify in the revised manuscript that for the age-based multi-group analysis, puberty status was determined using the parent-reported puberty onset variable from the HBN pre-interview questionnaire. This resulted in a split of the sample at 10.84 years of age, which reflects the mean reported onset of puberty in the dataset. We considered alternative approaches (e.g., applying North American average puberty onset age), but discarded these options since the sample is not exclusively representative of North American children and adolescents. As no hormonal data were available, we deemed the parent-reported onset variable to be the most objective indicator in this dataset. For these reasons, we did not undertake further changes in this section, i.e., we did not change the method of determination of puberty onset. We clarify our chosen method in the manuscript:

“For the age-based MGA, the mean age of puberty onset in the children and adolescents was used, resulting in a split of the sample at 10.84 years of age. This parent-reported puberty onset variable was taken from HBN’s pre-interview questionnaire.”

**Comment #9:** *“L278 Concerning variables that survived, was removal sequential (e.g. size of t value) or were decisions being made by humans?”*

**Response #9:** Thank you for this important point regarding statistical transparency. The variable selection process combined automated and criteria-based steps. Specifically, piece-wise SEM is a data-driven iterative process, and lasso regression was used to indicate which variables to retain. Thus, removal was sequential in that poorly performing predictors were excluded step by step. At the same time, inclusion in the final model required manual selection of objective criteria: only variables with p < 0.001 and an effect size *β* > 0.1 were retained. We have clarified this procedure in the revised manuscript to ensure greater transparency:

Variable selection thus combined automated and criteria-based steps: poorly performing predictors were sequentially removed through the iterative SEM process, and only variables that survived lasso regularization and met the thresholds of p < 0.001 and *β* > 0.1 were retained in the final model.

**Comment #10:** *“L292 Do you mean "samples" rather than "sample" if two halves?”*

**Response #10:** Thank you for catching this. In this context, we were referring to a comparison of the complete HBN sample (first column of Table 1) with the two stratified subsets (DS1 and DS2). This phrasing was intended to convey that no bias was introduced into the dataset halves that could affect the EFA and CFA results. We have revised the wording in the manuscript to make this clearer:

Sample characteristics are shown in Table 1 (see above). Age, sex, and race distributions remained similar between the complete sample and the two stratified halves, *DS1* and *DS2*.

**Comment #11:** *“L307 Is "variance in the data" the right phrase? Variance in items?”*

**Response #11:** Thank you, we agree that this seems imprecise. We have now made a small change for correctness:

This model explained 47.6% of the total variable variance utilizing the WLSMV estimator […].

**Comment #12:** *“L311 p-value = 1?.”*

**Response #12:** Understandable why this seems odd. R gives a p-value for test of close fit for RMSEA (“p-close”), which tests H0: RMSEA ≤ 0.05. So a large p-value here means that the model is consistent with a “close fit”, meaning that this value of RMSEA ≤ 0.05 is pretty certain. The value of 1 is rounded up from 0.999 by R, but we have now changed it to “>0.999” in Table 3 and 4 (and their footers), as well as in relevant parts of the entire manuscript:

Looking at the scaled fit measures, the model revealed a scaled χ^2^ value of 11885.117 (p-value < 0.001) with 2697 df, a CFI = 0.923, TLI = 0.914, SRMR = 0.063, and RMSEA = 0.041 (CI = [0.040;0.042], with the test of close fit (H_0_: RMSEA ≤ 0.05) yielding p > 0.999 see Table 3).

**Comment #13:** *“L327 "In the supplementary" - incomplete sentence.”*

**Response #13:** Perhaps the version uploaded to the submission portal the text got shifted somehow, but in our file the sentence states: “In the supplementary, a path diagram with the latent factors and all loading variables can be inspected (Figure S1).” We indicate where the reader may find the typical factor structure illustration in the supplementary. In the newly uploaded version of the edited manuscript, this sentence should be present.

**Comment #14:** *“L442 The postpubescent data fitting better is unsurprising, because several constructs are more likely to be expressed more strongly here e.g. externalising, sexual tendencies in later years. This should be a discussion point.”*

**Response #14:** Thank you for the comment. We agree that certain constructs, such as externalizing and sexual preoccupations, may be expressed more strongly in later adolescence. However, in our data the observed fit difference between pre- and postpubescent groups was quantitatively negligible (e.g., CFI = 0.907 vs. 0.908; RMSEA = 0.042 vs. 0.043). For this reason, we refrained from emphasizing the difference, as doing so would appear somewhat contradictory to our conclusion regarding overall model robustness across age groups. Moreover, the items related to sexual tendencies represented only a very small proportion of the included variables, and their factor loadings did not differ meaningfully between pre- and postpubescent participants.

**Comment #15:** *“L675 I was puzzled by this statement because surely any CA is a statistical summary of covariance of its items? This would apply to any model not just bifactor or g.”*

**Response #15:** Thank you for this observation, which indeed requires clarification. We agree that any factor model can be described as a statistical summary of item covariances. The sentence in question was part of a broader argument concerning critiques of bifactor models: specifically, that the emergence of a strong general g factor in bifactor modeling is often interpreted as a statistical artifact, more so than in other factor-analytic approaches. This nuance may not have been sufficiently clear in the original text. We have therefore revised the manuscript to emphasize this distinction more explicitly.

For example, the additional variance captured by a bifactor may not be theoretically meaningful (i.e., is residual covariance) and “only a statistical summary of the covariance among psychopathological symptoms” [101]. In other words, the general factor in bifactor models has a propensity to reflect residual covariance among items, which is statistically strong but theoretically ambiguous. As a result, it may represent a statistical artifact rather than a meaningful dimension.

**Comment #16:** *“L696 Here and elsewhere, why the emphasis on internalising but not externalising symptoms - the PID-5 covers both.* [*https://psycnet.apa.org/record/2019-70135-001*](https://psycnet.apa.org/record/2019-70135-001)*.”*

**Response #16:** Thank you for this thoughtful question. We chose to focus on the internalizing spectrum as a first step to evaluate the applicability of factor-analytic methods and to test the compatibility of the HiTOP model in childhood and adolescence. Given the high prevalence of DSM-based externalizing diagnoses in our sample (>60%), we considered it particularly important to examine internalizing symptoms, as these are often underdiagnosed in some populations (e.g., boys and young men) despite high rates of comorbidity. Our findings demonstrate that even in children and adolescents with externalizing diagnoses, a coherent structure of internalizing symptoms emerges.

We note, however, that we are also conducting ongoing work assessing the fit of the complete HiTOP spectrum in these age groups which is not limited to the internalizing domain. Unfortunately, the PID-5 was not included in the HBN test battery and therefore was unavailable for our analyses.
