# Supplementary material for: Toward dimensional psychiatry in youth: A data-driven analysis of transdiagnostic internalizing symptoms in childhood and adolescence
Source: PLOS Ment Health. 2025 Dec 17;2(12):e0000509. doi: 10.1371/journal.pmen.0000509 (PMC12798457; doi:10.1371/journal.pmen.0000509)
Supplement: Attachment — Submitted filename: RebuttalLetter_Engler.docx [file pmen.0000509.s005.docx]

Manuscript Number: PMEN-D-25-00214

Title: Toward Dimensional Psychiatry in Youth: A Data-Driven Analysis of Transdiagnostic Internalizing Symptoms in Childhood and Adolescence

Zurich, 21.10.2025

**Re-submission**

Dear Prof. Dr. Cardona,

We are happy to see that our changes following the reviewer’s recommendations were satisfactory. We also appreciate the second reviewer’s thoroughness, time and effort through a second reading of our paper after the first round of reviews.

As with the previous resubmission, we have quoted and put reviewer comments in italics, whereas extracts of the main manuscript and adaptations in accordance with the reviewer's remarks are in blue in this rebuttal letter. Once again, we have highlighted all new changes in the main manuscript.

We appreciate the opportunity to re-resubmit our manuscript following one reviewer’s additional comments. With these additional changes, we believe to have addressed all necessary adjustments and comments. In addition, we are confident that our paper now better adheres to quality standards and journal requirements for PLOS Mental Health.

On behalf of the co-author,

Sincerely,

Ines M. Engler

**Reviewer #1:**

*I believe this article is well-written and sufficiently addresses the topic!*

**Reviewer #2:**

*This is a good quality revision which addresses my previous concerns and suggestions. It is a nice paper and will open doors for the study team to do further papers using these constructs.*

*I have done a second fresh reading - apologies if I raise new things, I do not intend to generate extra work, but often read things differently on a resubmission. I have only minor suggestions below.*

**Comment #1:** *However, I think the results section needs cutting down at this point - there is far too much detail, and every decisions is described rather than the most important ones. It is good the analysis was diligently reported in full, and striking a balance is something we all struggle with, but it currently reads too much like a thesis rather than a journal article. Look at similar published papers and they are more concise. It is OK to move material to appendices, or just keep a record of less substantive decisions made in case asked by readers. Now it has been peer reviewed, we can trust the work has been done to a high standard, and lose some of the text. Perhaps the editor can make a judgement.*

**Response #1:** We thank the reviewer for this helpful comment and fully agree that the Results section should be more concise and focused on the key findings. We have substantially shortened this section (from 2204 to 1075 words) to focus on the main outcomes and essential procedural steps. This streamlining removes excessive detail and descriptive decision steps, in line with the reviewer’s suggestion. We believe that, together with the detailed Methods and Supplementary Materials, the Results now remain clear and interpretable.

**Comment #2:** *L132 Check journal requirements but I usually add commas to thousands e.g. 4,142 (here and throughout). Proofread for consistency throughout (and decide on approach in all tables, consistency needed).*

**Response #2:** We have now added commas to thousands in all pertinent numbers in tables and throughout the text.

**Comment #3:** *L153 If known, I suggest added the % whose parents consented, but the participant did not assent and were excluded. It is good practice to report every step in which participants can be excluded (e.g. in flow diagram) and useful to understand if there were any differences here. Not essential, but if you have this information I would included it.*

**Response #3:** We thank the reviewer for this thoughtful suggestion and agree that including this information would enhance transparency in reporting sample selection. Unfortunately, the Healthy Brain Network (HBN) dataset does not provide details on the proportion of cases in which parental consent was given but participant assent was not obtained.

**Comment #4:** *Table 1 - do not use 3 decimal places if 2 for other variables (I would suggest 1 throughout - nicer presentation and avoids pseudo-precision)
Table 1 - I strongly recommend never using dashes "-" in tables or when presenting confidence intervals, because these can be confused with minus signs. Use "to" word, or "," comma. You have used semicolon in Table 3 which is OK.*

**Response #4:** Thank you for the indications. We have removed all hyphens between numbers from tables (e.g. *age 5 to 21* in table 1 and *scale 0 to 2* in table 2). In addition, we have rounded numbers throughout to 2 decimal places instead of 3; with two exceptions for precision:

- “p-value < 0.001”: we deem this important transparency in statistical significance
- RMSEA CIs: the CIs are so narrow that they would include the same number multiple times, i.e., RMSEA = 0.041 [0.040;0.042] would be 0.04 [0.04;0.04] when reducing to two decimal spaces. This would create a loss of precision and may be confusing to the reader. An example for this can be seen in table 3.

**Comment #5:** *[P9 You may wish to explore, in future research, Mplus, which can do EFA/CFA with missing data quite well. It can also do "exploratory CFA" in one set of models. It is expensive but handles these sorts of scenarios nicely.]*

**Response #5:** Thank you for the recommendation. We will happily keep this in mind for any future work with factor analysis.

**Comment #6*:*** *L314 Be careful with "outcomes" which can imply causality. Even in longitudinal data, causality is not straightforward. Sometimes it is better to report the construct and the age (e.g. distress at age 18 or whatever) in case readers question bidirectional effects, reverse causation, residual confounding, time-varying confounding etc. In cross-sectional it is better not to say "outcomes" at all.*

**Response #6:** We thank the reviewer for this important observation. While we had clarified in the Discussion that we do not intend to make any causal inferences and that our results reflect statistical rather than temporal dependencies, we have now replaced the term “outcomes” with “indicator” (or “quality of life indicator”, since our “outcome” variable is a construct reflecting daily functioning; see *WHODAS*) to avoid any implications of causality. Accordingly, we have removed all mention of this limitation in the *Discussion* section.

**Comment #7:** *L339 Sequentially according to what? t value size?*

**Response #7:** We indicate the criteria at the end of the sentence (“*p < 0.001 and β > 0.1*”), but acknowledge now that the link to the sequential removal may not have been evident in the original sentence. We have adapted and extended the sentence accordingly:

“Variable selection thus combined automated and criteria-based steps: poorly performing indicators were sequentially removed through the iterative SEM process, i.e., based on p < 0.001, *β* > 0.1, and lack of model fit improvement. Only variables that survived lasso regularization and met the significance and effect size thresholds were retained in the final model.”

**Comment #8:** *L372 Again here and elsewhere, 3 decimal places is not necessary*

**Response #8**: Adapted.

**Comment #9:** *L822 This is too apologetic - we all have to label constructs, in order to study and talk about them to further understanding. I would tone this down. It is the reliability/validity that will be important longer term, no one should get upset about the names particularly. We could spend our lives debating names - focus on their practical utility, predictive validity (the ultimate test).*

**Response #9:** We thank the reviewer for this insightful comment. We have revised and shortened this discussion point following this recommendation. Our concern was that readers might misinterpret the divergence between our labels to HiTOP’s as implying superiority of our terminology, which we do not intend. The revised paragraph now emphasizes that the observed differences highlight the need to refine HiTOP for younger populations:

“A further limitation is the labeling of our factors. We adopted HiTOP’s *Distress* label for one of our latent factors due to high overlap of its associated psychopathological constructs. Our other factors did not directly align with HiTOP’s, as the model does, for example, not include a factor explicitly covering obsessions, compulsions and behavioral disinhibitions such as our *OC* factor. To this end, we adopted our own labels for three latent factors, suggesting that the structure of psychopathology in childhood may differ from that in adulthood.”

We also expanded the on the practical, particularly clinical, utility of these factors and factorial models in the Discussion section.

**Comment #10:** *L1244 There are 109 references - that is very high for a journal article (see above). You could lose anything up to a half I think.*

**Response #10:** We have reduced the references from 109 to 76 (reduction of ≈30%). We are certain that this strikes a better balance, while ensuring that necessary references for statistical methods, choices of thresholds, and contextualization of our results remain available to the reader.

**Comment #11:** *Figure 1 - this should ideally separately show the entire eligible population, approached, recruited, consented, assented (if available). See CONSORT/STROBE guidelines. Clinicians are trained in critical appraisal (e.g. CASP) and this is one of the things they will scrutinize in detail - who are the population, who are the sample, are they different, in what ways? At every step.*

**Response #11:** We agree that this is interesting and highly relevant information. Unfortunately, the HBN once again does not include this information. Additionally, it is a public dataset and participants are not recruited, as it works on a self-referred basis.

**Comment #12:** *To Editor: I was not able to check the data and code availability at* [*https://osf.io/br8wd/files/osfstorage*](https://osf.io/br8wd/files/osfstorage)*. It was being upgraded at the time of writing ("We are verifying the upgrade to the OSF frontend codebase. Please refrain from using OSF until you see this message removed"). Perhaps you want to check availability before your decision.*

**Response #12:** Albeit the comment not being directed at us, we have once again checked our OSF project, and can ensure the availability of the described materials. Luckily, OSF is now accessible again after the update.
